# The Leading Transplantation Journals: A Trend Analysis, 2011–2021

**DOI:** 10.1155/2023/8858320

**Published:** 2023-09-26

**Authors:** Badi Rawashdeh, Saif Aldeen Alryalat, Joohyun Kim, Calvin Eriksen, Mohammad Abu Assi, Raj Prasad, Matthew Cooper

**Affiliations:** ^1^Division of Transplant Surgery, Medical College of Wisconsin, Milwaukee, WI, USA; ^2^Faculty of Medicine, Jordan University, Amman, Jordan

## Abstract

**Background:**

As the field of transplantation has expanded, so have the quantity and variety of articles published on the topic. Evaluation of publications and journals is crucial to the expansion of transplant research. This study investigated the research output and journal metrics of the leading solid organ transplant journals published between 2011 and 2021 based on estimations of the trends in the category CiteScore from the Scopus database.

**Materials and Methods:**

We obtained data on the listed journals from the Scopus Source List. We then filtered the list for “Transplantation” journals. Only the top quartiles or quartile 1 (Q1) journals were placed in this category. This study focused specifically on transplantation journals and did not include other journals related to diseases of transplanted organs such as the kidney, liver, heart, and lungs.

**Results:**

The number of transplantation journals increased by 42.8% in the last ten years, from 28 in 2011 to 40 in 2021. Between 2011 and 2021, nine transplantation journals ranked in the highest quartile (Q1). The American Journal of Transplantation was the top journal in both years, with a 150% increase in citations and an 11.2% increase in articles published. Open access (OA) transplant journals rose from 3 in 2011 to 10 in 2021. In 2021, OA journals earned 8,555 citations, a 125% increase from 2011. Despite this increase, non-OA journals received more citations than OA in 2021 (*p* value 0.026).

**Conclusion:**

Solid organ transplantation advances lead to more publications and citations. Regular journals and publications evaluation benefits academics and policymakers by promoting the growth of research. This study examined solid organ transplantation journals and gave a global perspective on transplant journal rankings and compared their status in 2011 and 2021.

## 1. Introduction

During the early twentieth century, a number of eminent researchers pioneered the concept of transplantation and the fundamental principles of transplantation immunology [1]. Since then, the development of organ transplantation has become one of the great tales of medicine. Along with the other health professions and sciences, the field of transplantation began to review its activities and accomplishments methodically. The progression in the field of transplantation was reflected in the nature of research material to the point where there was an adequate awareness of the need for new journals that specialized in transplant subjects, which, since their establishment, have contributed to the advancement of the field, a contribution accompanied by an increase in the number of publications and citations.

The total number of journal articles and citations is considered an indicator in international scientific rankings and a measure of a field's academic productivity [2]. Continual assessment of journals and publications is essential to the growth and maintenance of the research enterprise since it provides invaluable insight to researchers and policymakers. The most prevalent journal-based metrics are CiteScore and Impact Factor (IF), both of which are crucial for determining how well a journal has performed over time [3]. IF has grown in popularity as a metric of a journal's quality. However, the more recent metric, the CiteScore, provides a more comprehensive, transparent, and up-to-date representation of a journal's influence than a single measure alone [3–5].

The progress in academic performance of transplantation journals has rarely been studied. An objective and multiperspective evaluation of the academic performance of transplant journals might provide a complete understanding of the recent evolution of transplant scientific studies. In this study, we assessed research output and journal metrics among top-performing solid organ transplant journals. We compared academic performance from 2011 to 2021 by using estimates for the trends in the category CiteScore from the Scopus database. Furthermore, we examined the open access (OA) status of transplant journals and the effect of that status on different journal indexes.

## 2. Methodology

We collected data on the included journals from the Scopus Source List on November 20, 2022, which is a list of journals and their respective metrics data since 2011. Journals are classified into subfields based on the aims and scope of the title and on the content they publish.

We filtered the list for “Transplantation” journals. Scopus classifies journals according to the All Science Journal Classification Codes (ASJC), a coding system that is provided by the Scopus database and is also summarized in Dataset [6]. ASJC classifies journals into four main subject areas: health sciences, life sciences, physical sciences, and social sciences and humanities. Each subject area is also classified into different subcategories. The health sciences area is classified into 29 subcategories, one of which is “Medicine,” which is further classified into 49 disciplines, including “Transplantation.” We initially searched all solid organ transplant journals included in all quartiles, Q1, Q2, Q3, and Q4, and then included just the top quartiles, or quartile 1 (Q1) journals, which are defined as the first position of the top 25% of journals in a certain subject, placed in this category. Nonsolid organ transplant journals, such as those specializing in bone marrow transplantation, were excluded.

For this study, we focused on evaluating transplant journals published between 2011 and 2021 using the Scopus database and the CiteScore metric. We did not evaluate and compare the impact factor (IF) based on the Web of Science database for several reasons. First, Scopus provides a larger database with broader coverage, as it indexes more journals than Web of Science [2]. This wider coverage allows for a more comprehensive evaluation of the transplant journals. Second, Scopus has a more transparent calculation methodology for its CiteScore metric compared to the IF, making it a more reliable and easily verifiable measure of journal performance [3, 7]. Furthermore, Scopus provides additional journal metrics such as the source normalized impact per paper (SNIP) and the SCImago journal rank (SJR), which can offer a more well-rounded assessment of journal performance [2]. By focusing on the Scopus database and its associated metrics, we aimed to provide a robust and transparent evaluation of the academic performance of transplant journals.

### 2.1. Variables and Definitions

For each journal, we extracted the following variables:

- CiteScore: it measures average citations received per document published in the serial.
- CiteScore Percentile: it indicates the relative standing of a serial title in its subject field. For example, a serial that has a CiteScore percentile of 96% is ranked according to CiteScore as high or higher than 96% of titles in that category. A title will receive a CiteScore percentile for each subject area in which it is indexed in Scopus.
- Citation count: citations received in one year (e.g., 2021) for the documents published in the previous 3 years (e.g., 2019–2021).
- Scholarly output: sum of documents published in the serial title (e.g., 2021) in the 3 years prior to the year of the metric (e.g., 2019–2021).
- Percent Cited: the proportion of the documents (e.g., 2018–20) that have received at least 1 citation (e.g., 2021).
- SCImago Journal Rank (SJR): it measures weighted citations received by the serial. Citation weighting depends on subject field and prestige (SJR) of the citing serial.
- Source Normalized Impact per Paper (SNIP): it measures actual citations received relative to citations expected for the serial's subject field.
- SCImago Quartiles: quartile 1 = 99th–75th CiteScore percentile. Quartile 2 = 74th–50th CiteScore percentile. Quartile 3 = 49th–25th CiteScore percentile. Quartile 4 = 24th–0 CiteScore percentile.

OA Journals covered by Scopus are indicated as “Open Access” if the journal is listed in the Directory of Open Access Journals (DOAJ) and/or the Directory of Open Access Scholarly Resources (ROAD).

### 2.2. Statistical Analysis

We used IBM SPSS Statistics for Windows, version 26.0 (IBM Corp., Armonk, N.Y., USA), in our analysis. We used the mean (±standard deviation) to describe continuous variables. We used to count (frequency) to describe other nominal variables. We performed an independent sample *t*-test to analyze the mean difference between O.A. and non-OA journals and between years with each continuous measurement (e.g., citation and document), and we presented the data in mean difference and standard deviation (±). We adopted a *p* value of 0.05 as a significant threshold.

## 3. Results

The number of transplantation journals increased by 42.8% in the last ten years, from 28 in 2011 to 40 in 2021. The yearly average number of articles published by the existing 28 journals in 2011 was 643.82 (±850.18). This is compared to the yearly mean article published by the existing 40 journals in 2021, 465.07 (±521.49), a difference that was not statistically significant (*p* = 0.287). The number of citations received in 2011 was 82,877 (mean: 2959.89 ± 4331.44) citations, while the number of citations received in 2021 was 95,130 (mean: 2378.25 ± 3862.07) citations, a 14.8% increase from 2011, with a nonstatistically significant difference (*p* value of 0.563).

Tables 1 and 2 show the top 25% quartile journals (Q1) in the field of transplantation between the years 2011 and 2021. There were nine journals ranked as top-quartile journals in the field of transplantation in 2011 and 2021. The top journal was “American Journal of Transplantation” in both years, which had a 150% increase in the number of citations received, compared to only an 11.2% increase in the number of documents published.

**Figure 1:**
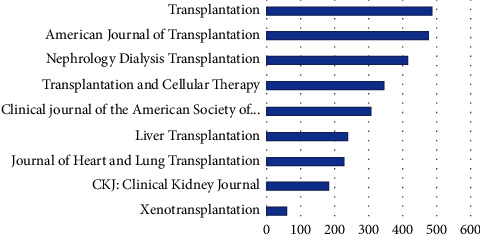
Mean number of articles published per year by Q1 journals in 2021 since 2011.

Transplantation publishes the most articles with an average of 490 every year, followed by the American Journal of Transplantation with an average of 478 per year, as demonstrated in Figure 1, which depicts the average number of articles published per year by Q1 publications in 2021 since 2011. The Clinical Kidney Journal and xenotransplantation have the lowest number of publications with 180 and 60 articles per year, respectively.

### 3.1. CiteScore Trend

**Figure 2:**
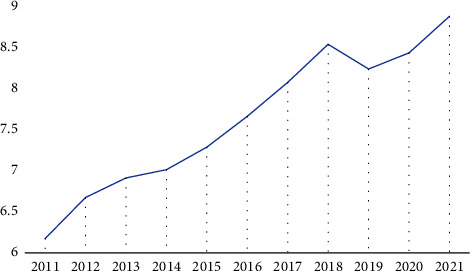
Mean CiteScore trend for the top quartile journals in 2021 since 2011.

Figure 2 shows the trend of the average CiteScore of all top-performing journals from 2011 to 2021. In 2011, the average CiteScore was 6.1, but it reached an all-time high of 8.8 in 2021.  Table 3 shows the change in top quartile journals in the year 2011 and the new rank change in the year 2021, along with the change in the number of citations received (proportional relation) and documents published (inverse relation). The drop in the number of citations received or the rise in the number of documents published without a matching rise in the number of citations would have a negative impact on the journal's ranking.

### 3.2. Open Access Journals

The number of transplant journals increased by 230% from 3 in 2011 to 10 in 2021. A total of 1,778 (mean 592.67 ± 38.07) documents were published in OA journals in 2011, compared to 3,022 (mean 302.2 ± 240.9) documents published in OA journals in 2021 (*p* = 0.104), representing a 70% increase in the quantity of documents. For citations, OA journals received 8555 citations in 2021, compared to 3801 in 2011, a 125% increase. Despite these improvements, the number of non-OA journals had significantly higher citations in 2021 compared to OA journals (*p* = 0.026), as shown in Table 4.

## 4. Discussion

Breakthroughs in the domains of immunology, immunosuppression, organ preservation, and surgical techniques led to increased research output and the necessity for journals devoted to the field of transplantation. In 1966, the first transplantation-specific journal was established by the Transplantation Society and was named Transplantation [6]. As the field as a whole progressed, more specialized journals emerged, such as those for infectious disease related to transplantation (transplant infectious disease), pediatrics (pediatric transplantation), and xenograft transplantation (xenotransplantation). In our analysis, we observed an almost 40% increase in the number of transplantation journals between 2011 and 2021. This was also associated with an increase in the number of OA journals by 230% from 3 in 2011 to 10 in 2021. We observed that the top journals are still non-OA journals, and their citation indices increased, mostly driven by the articles published by the new journals.

**Figure 3:**
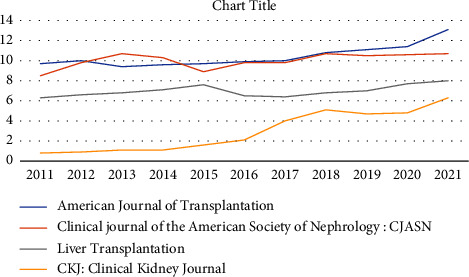
CiteScore trend of the American Journal of Transplantation, Clinical Journal of the American Society of Nephrology, Liver Transplantation and Clinical Kidney Journal since 2011.

Our findings demonstrating an increase in citation frequency in transplantation journals are consistent with this overarching trend, which has been observed in all areas of medical research [8, 9]. Upon further examination of the data, specifically focusing on Figures 2 and 3, an apparent rise in citations can be observed starting from the year 2016. Significantly, the implementation of machine perfusion in several solid organs has likely stimulated a substantial amount of scholarly investigation, resulting in a notable increase in the number of published academic articles [10, 11]. The years 2020 and 2021 had an unparalleled increase, potentially linked to the abundance of papers related to COVID-19, which extensively occupied numerous journals, thus augmenting both the quantity and references of publications [12].

Nevertheless, it is important to make a crucial differentiation: an increasing quantity of articles and citations does not necessarily indicate an improvement in the quality of research. Upon reflecting on the environment of academic research a few decades ago, it becomes evident that there was a lower quantity of papers being published, while the ones that were produced tended to be more innovative and influential in their respective fields. Contrarily, modern medical research, which includes transplants, frequently confirms or reiterates findings from earlier studies, making genuinely original innovations scarce. Moreover, while OA has transformed academic publishing by making research more accessible, its article processing charge model has inadvertently paved the way for predatory journals. [13]. These journals utilize the “pay-to-publish” model without maintaining academic standards [14]. It is necessary to differentiate between legitimate OA journals that adhere to rigorous academic standards and those that exploit the system. Researchers can identify and avoid predatory journals with the aid of the Directory of Open Access Journals (DOAJ) and guidelines from reputable organizations [15]. Fortunately, none of the journals assessed in this study are representative of this problematic trend.

There is a growing consensus that a system to grade the quality of scientific journals is essential, given the vast quantities of publications across all the major scientific disciplines, and transplant journals are no exception. Scopus (Elsevier®) and Web of Science (Clarivate®) are two of the most prominent journal databases, and they established two methods to assist with the ranking of scientific journals. CiteScore, which is a product that was introduced by Elsevier in order to compete with IF, which is a product that was released by Clarivate Analytics (formerly part of Thomson Reuters®) [3].

The development of medical journals' academic performance has already been examined in a number of medical specialties [16–20] with the discipline of radiology demonstrating that all impact indices witnessed a modest increase between 2010 and 2019 [16]. Another study found that the number of emergency medicine journals increased by 58% between 2000 and 2009 and that, since 2000, the impact indices for all emergency medicine journals have been trending upward [20]. As with journals in other fields, transplant journals have strived to raise their impact measures by increasing the number of citations they obtain or the number of articles they publish.

Both the CiteScore and IF use the total number of publications and citations for their calculations [7, 21]. The two metrics differ in that IF is less open about how it was calculated than the CiteScore [22]. The Journal IF has been deemed questionable because the calculations are “based on undisclosed data” despite the fact that the database has become more transparent recently [22, 23]. Even high-impact journals have criticized the quality of the data used to calculate the IF [4]. In our study, we opted for CiteScore because it has several advantages over Impact Factor. CiteScore considers a broader range of document types, including articles, reviews, conference papers, and book chapters, resulting in a more comprehensive assessment of a journal's impact [2, 24]. In addition, CiteScore is based on a three-year citation window, which provides a more stable metric compared to the two-year window of Impact Factor [24].

Furthermore, CiteScore makes it possible for any Scopus subscriber to instantly access both the citing and cited documents [3]. For example, CiteScore in 2020 counts the citations received in 2017–2020 for articles, reviews, conference papers, book chapters, and data papers published in 2017–2020 and divides this by the number of publications published in 2017–2020 [24]. As a result, we believe that CiteScore offers a better representation of a journal's performance.

CiteScore measures are included in the Scopus bundle of journal metrics, which also includes SNIP (Source Normalized Impact per Paper), SJR (SCImago Journal Rank), citation- and document-counts, and citation percentage [24]. Many institutions rank journals with Scopus bibliometric indicators to evaluate the track record of scholars seeking hire or promotion; these metrics are also used to allocate financial bonuses or to evaluate funding applications [25].

As can be seen in Figure 3, the CireScore of the American Journal of Transplantation, the Clinical Journal of the American Society of Nephrology, Liver Transplantation, and the Clinical Kidney Journal have all been on an upward trajectory every year since 2016. Xenotransplantation entered the Q1 club in 2016 with a CireScore of 6.4, and it has been there ever since, with a CireScore of 6.1 in 2021. In the 2021 list, Transplantation Review, Transplant International, and Current Opinion in Organ Transplantation were substituted for Transplantation and Cellular Therapy, Clinical Kidney Journal, and Xenotransplantation.

In contrast to the old paradigm of scientific publishing, which required users to pay to access articles, OA publication is a relatively new phenomenon in the research community and represents a form of development in a field [17]. OA has revolutionized scientific publishing by making research available to more readers [17, 26]. OA articles are published more quickly and can be shared online, accelerating the availability of new knowledge and the pace of scientific research [17, 27].

According to numerous studies conducted in a wide variety of medical fields, OA medical journals have significantly higher CiteScores, percent cited articles, and SNIP compared to non-OA journals [17, 26, 28, 29]. However, for the field of transplant our analysis revealed that, in 2021, subscription journals significantly outperformed OA transplant journals in terms of citations (*p* value 0.026); number of articles; CireScore; SNIP; and SJR. The dominance of the subscription journal in the transplantation field may be due to the preference of authors with unique and influential (i.e., more citable) outcomes to submit their findings to prestigious, mostly subscription-based journals. Although the number of OA transplant journals tripled between the years 2011 and 2021, our findings showed that eight of the top nine Q1 transplant journals in the Scopus ranking are paid-only journals that require a subscription to access. The Clinical Kidney Journal was the only OA journal to appear on the Q1 list in 2021, while Transplant International was the only one that was on the Q1 list in 2011.

It is important to note that articles in the field are also published in nonspecialty journals. We used the Scopus database for our analysis, as it is the most inclusive, high-quality database [2]. However, other databases may provide additional information on the field and should be considered in future research.

## 5. Conclusion

The increase in the number of articles and citations in the field of transplantation and the rise in the number of specialized journals reflect the progress established in the field. Evaluation of journals and publications on a regular basis is crucial to the growth and maintenance of research enterprises because of the invaluable insight it provides to academics and policymakers. This study analyzed journals that specialize in solid organ transplantation and provided a comprehensive overview of their development. It provided a global perspective on the advancement of transplant journal rankings and compared the status of the journals in 2011 and 2021.

## Figures and Tables

**Table 1:** Top quartile (Q1) journals in 2011.

Journal	Open access	CiteScore	Citations^*∗*^	Documents^*∗*^	Percent cited	SNIP	SJR	Publisher
American Journal of Transplantation	No	9.7	12417	1278	82	2.05	3.23	Wiley-Blackwell
Clinical journal of the American Society of Nephrology	No	8.5	9813	1154	82	2.21	2.66	American Society of Nephrology
Journal of Heart and Lung Transplantation	No	6.9	5616	809	78	1.55	2.69	Elsevier
Transplantation	No	6.9	12687	1836	82	1.35	1.85	Wolters Kluwer Health
Nephrology Dialysis Transplantation	No	6.4	14294	2229	82	1.43	1.51	Oxford University Press
Transplantation Reviews	No	6.4	636	99	82	1.31	1.34	Elsevier
Liver Transplantation	No	6.3	5225	836	80	1.46	1.43	Wiley-Blackwell
Transplant International	Yes	4.9	2721	556	78	1.11	1.3	European Society for Organ Transplantation
Current Opinion in Organ Transplantation	No	4.7	1966	417	75	1.04	1.31	Wolters Kluwer Health

^*∗*^From the total number for the period of 2008–2011.

**Table 2:** Top quartile (Q1) journals in 2021.

Journal	Open access	CiteScore	Citations^*∗*^	Documents^*∗*^	Percent cited	SNIP	SJR	Publisher
American Journal of Transplantation	No	13.1	18648	1421	89	2.33	3.31	Wiley-Blackwell
Journal of Heart and Lung Transplantation	No	11.5	6036	524	83	3.2	2.47	Elsevier
Clinical journal of the American Society of Nephrology	No	10.7	8435	791	88	2.31	2.34	American society of nephrology
Nephrology Dialysis Transplantation	No	8.8	9848	1120	86	1.81	1.54	Oxford university press
Liver Transplantation	No	8	4314	540	87	1.77	1.61	Wiley-Blackwell
Transplantation and Cellular Therapy	No	7.8	10088	1288	81	1.44		Elsevier
Transplantation	No	7.6	7434	981	76	1.6	1.41	Wolters Kluwer Health
Clinical Kidney Journal	Yes	6.3	3351	528	83	1.66	1.34	Oxford University Press
Xenotransplantation	No	6.1	1442	235	89	0.86	0.8	Wiley-Blackwell

^*∗*^The total number for the period of 2018–2021.

**Table 3:** Top quartile (Q1) journals in 2011 and the change in their rank in 2021 ranking.

Journal	New ranks	Citation change (%)	Document change (%)
American Journal of Transplantation	Same	+50	11
Clinical journal of the American Society of Nephrology	Dropped by 1 rank	−14	−31
Journal of Heart and Lung Transplantation	Promoted by 2 ranks	+7	−35
Transplantation	Dropped by 3 ranks	−41	−47
Nephrology Dialysis Transplantation	Promoted by 2 ranks	−31	−50
Transplantation Reviews	Dropped by 6 ranks	+13	32
Liver Transplantation	Promoted by 2 ranks	−17	−35
Transplant International	Dropped by 3 ranks	−1.70	12.90
Current Opinion in Organ Transplantation	Dropped by 4 ranks	−29.20	−6.20

^*∗*^A positive sign means an increase from 2011 to 2021 and a negative sign means a decrease from 2011 to 2021.

**Table 4:** Comparison between open access (OA) and non-OA journals in 2021 ranking.

	Open access journal				*p* value
	No		Yes		
	Mean	Standard deviation	Mean	Standard deviation	
Citations	2885.83	4306.66	855.50	1198.20	0.026
Documents	519.37	579.23	302.20	240.99	0.104
CiteScore	3.86	3.78	2.02	2.06	0.062
SNIP	0.92	0.79	0.60	0.54	0.176
SJR	0.79	0.82	0.52	0.45	0.254

## References

[1] Barker C. F., Markmann J. F. (2013). Historical overview of transplantation. *Cold Spring Harbor Perspectives in Medicine*.

[2] AlRyalat S. A. S., Malkawi L. W., Momani S. M. (2019). Comparing bibliometric analysis using PubMed, scopus, and Web of science databases. *Journal of Visualized Experiments: JoVE*.

[3] Fernandez-Llimos F. (2018). Differences and similarities between journal impact factor and CiteScore. *Pharmacy Practice*.

[4] Errors in citation statistics (2002). Errors in citation statistics. *Nature*.

[5] Liu X. L., Gai S. S., Zhou J. (2016). Journal impact factor: do the numerator and denominator need correction?. *PLoS One*.

[6] Scopus (2022). Access and use support center. https://service.elsevier.com/app/answers/detail/a_id/14882/supporthub/scopus.

[7] Brown T., Gutman S. A. (2019). Impact factor, eigenfactor, article influence, scopus SNIP, and SCImage journal rank of occupational therapy journals. *Scandinavian Journal of Occupational Therapy*.

[8] Fernandez-Guerrero I. M., Martin-Sanchez F. J., Burillo-Putze G., Graham C. A., Miro O. (2019). Analysis of the citation of articles published in the European Journal of Emergency Medicine since its foundation. *European Journal of Emergency Medicine*.

[9] Lerman K., Yu Y., Morstatter F., Pujara J. (2022). Gendered citation patterns among the scientific elite. *Proceedings of the National Academy of Sciences of the U S A*.

[10] Pezzati D., Pieroni E., Martinelli C. (2019). Liver machine preservation: state of the art. *Current Transplantation Reports*.

[11] Patrono D., De Stefano N., Martins P. N., Romagnoli R. (2022). Highlights from the Turin international workshop on liver machine perfusion. *Artificial Organs*.

[12] Rawashdeh B., AlRyalat S. A., Abuassi M. (Jun 2023). Impact of COVID-19 on abdominal organ transplantation: a bibliometric analysis. *Transplant Infectious Disease: An Official Journal of the Transplantation Society*.

[13] O’Kelly F., Fernandez N., Koyle M. A. (2019). Predatory publishing or a lack of peer review transparency?-a contemporary analysis of indexed open and non-open access articles in paediatric urology. *Journal of Pediatric Urology*.

[14] Tardif P. A., Mercier E., Moore L. (2021). Differentiating between questionable and legitimate trauma journals: a systematic review and evaluation of two sets of criteria. *Injury*.

[15] Cotter R. R., Funk L. M., Wong S. L. (2023). A review and assessment of open access surgery journals. *Journal of Surgical Research*.

[16] Fornell-Perez R., Merino-Bonilla J. A., Morandeira-Arrizabalaga C., Marin-Diez E., Rovira A., Ros-Mendoza L. H. (2021). A bibliometric study of the journal Radiologia during the period 2010-2019. *Radiología*.

[17] AlRyalat S. A., Saleh M., Alaqraa M. (2019). The impact of the open-access status on journal indices: a review of medical journals. *F1000Research*.

[18] Nielsen M. B., Seitz K. (2016). Impact factors and prediction of popular topics in a journal. *Ultraschall in der Medizin*.

[19] Jones A. W. (2007). The distribution of forensic journals, reflections on authorship practices, peer-review and role of the impact factor. *Forensic Science International*.

[20] Lee C. H., Shih C. P., Chang Y. C., Chaou C. H. (2011). The evolution of academic performance in emergency medicine journals: viewpoint from 2000 to 2009 journal citation reports. *Academic Emergency Medicine*.

[21] Kavic M. S., Satava R. M. (2021). Scientific literature and evaluation metrics: impact factor, usage metrics, and altmetrics. *Journal of the Society of Laparoendoscopic Surgeons*.

[22] Mech E., Ahmed M. M., Tamale E., Holek M., Li G., Thabane L. (2020). Evaluating Journal Impact Factor: a systematic survey of the pros and cons, and overview of alternative measures. *Journal of Venomous Animals and Toxins including Tropical Diseases*.

[23] Rossner M., Van Epps H., Hill E. (2007). Show me the data. *The Journal of Cell Biology*.

[24] Baker D. W. (2020). Introducing CiteScore, our journal’s preferred citation index: moving beyond the impact factor. *Joint Commission Journal on Quality and Patient Safety*.

[25] Cortegiani A., Ippolito M., Ingoglia G. (2020). Citations and metrics of journals discontinued from Scopus for publication concerns: the GhoS(t)copus Project. *F1000Research*.

[26] Hua F., Sun H., Walsh T., Glenny A. M., Worthington H. (2017). Open access to journal articles in oncology: current situation and citation impact. *Annals of Oncology*.

[27] Lansingh V. C., Carter M. J. (2009). Does open access in ophthalmology affect how articles are subsequently cited in research?. *Ophthalmology*.

[28] Vadhera A. S., Lee J. S., Veloso I. L. (2022). Open access articles garner increased social media attention and citation rates compared with subscription access research articles: an altmetrics-based analysis. *The American Journal of Sports Medicine*.

[29] Yu X., Meng Z., Qin D., Shen C., Hua F. (2022). The long-term influence of Open Access on the scientific and social impact of dental journal articles: an updated analysis. *Journal of Dentistry*.

